# Links between looking and speaking in autism and first-degree relatives: insights into the expression of genetic liability to autism

**DOI:** 10.1186/s13229-018-0233-5

**Published:** 2018-10-10

**Authors:** Kritika Nayar, Peter C Gordon, Gary E Martin, Abigail L Hogan, Chelsea La Valle, Walker McKinney, Michelle Lee, Elizabeth S Norton, Molly Losh

**Affiliations:** 1 Northwestern University, Evanston, USA; 2 University of North Carolina at Chapel Hill, Chapel Hill, USA; 3 St. John’s University, New York City, USA; 4 University of South Carolina, Columbia, USA; 5 Boston University, Boston, USA; 6 University of Kansas, Lawrence, USA

**Keywords:** Rapid automatized naming, Language, Endophenotype, Autism spectrum disorder, Broad autism phenotype, Social communication, Restricted and repetitive behaviors, Eye movement, Gaze, Eye-voice span

## Abstract

**Background:**

Rapid automatized naming (RAN; naming of familiar items presented in an array) is a task that taps fundamental neurocognitive processes that are affected in a number of complex psychiatric conditions. Deficits in RAN have been repeatedly observed in autism spectrum disorder (ASD), and also among first-degree relatives, suggesting that RAN may tap features that index genetic liability to ASD. This study used eye tracking to examine neurocognitive mechanisms related to RAN performance in ASD and first-degree relatives, and investigated links to broader language and clinical-behavioral features.

**Methods:**

Fifty-one individuals with ASD, biological parents of individuals with ASD (*n* = 133), and respective control groups (*n* = 45 ASD controls; 58 parent controls) completed RAN on an eye tracker. Variables included naming time, frequency of errors, and measures of eye movement during RAN (eye-voice span, number of fixations and refixations).

**Results:**

Both the ASD and parent-ASD groups showed slower naming times, more errors, and atypical eye-movement patterns (e.g., increased fixations and refixations), relative to controls, with differences persisting after accounting for spousal resemblance. RAN ability and associated eye movement patterns were correlated with increased social-communicative impairment and increased repetitive behaviors in ASD. Longer RAN times and greater refixations in the parent-ASD group were driven by the subgroup who showed clinical-behavioral features of the broad autism phenotype (BAP). Finally, parent-child dyad correlations revealed associations between naming time and refixations in parents with the BAP and increased repetitive behaviors in their child with ASD.

**Conclusions:**

Differences in RAN performance and associated eye movement patterns detected in ASD and in parents, and links to broader social-communicative abilities, clinical features, and parent-child associations, suggest that RAN-related abilities might constitute genetically meaningful neurocognitive markers that can help bridge connections between underlying biology and ASD symptomatology.

## Background

Language impairments are a hallmark feature of autism spectrum disorder (ASD). Subtle differences in language have also been observed among first-degree relatives of individuals with ASD and have been described as part of the broad autism phenotype (BAP), or a set of subclinical personality and language features that mirror the core features of ASD in quality and are believed to reflect genetic liability to ASD [1–5]. Studies of the mechanisms underlying the clinical features of ASD and the BAP have the potential to reveal key markers of heritable risk to ASD. In this study, we investigated the coordination of gaze and language during a language processing task, rapid automatized naming (RAN), as a potential mechanism related to the clinical-behavioral features of ASD and the BAP.

RAN is a task that taps the fluency of cognitive and linguistic processes underlying complex language-related skills [6]. It involves quickly and accurately naming rows of common symbolic (letters/numbers) and non-symbolic (colors/objects) items presented in an array, and draws on a number of linguistic processes (e.g., lexical retrieval and connection of orthographic/written, semantic, and phonological/auditory representations), coordinates visual cues with vocal responses, and utilizes executive functions (e.g., working memory, capacity to maximize speed, regulating attention and avoiding interference between successive items) [7, 8]. Performance on RAN has been linked to a broad network of language-related brain regions (e.g., left-hemisphere language temporal areas (linguistic processes and semantic access), supplementary motor area and pre-motor area (articulation), the supramarginal gyrus (for grapheme-phoneme translation), right cerebellum (motor planning), and the visual cortex) [6, 9, 10] and is a strong predictor of reading skills [11].

Studies of eye gaze patterns during RAN have provided additional insights into the attentional, perceptional, and motor mechanisms supporting fluent RAN performance and are linked to clinical-behavioral outcomes. For instance, a specific eye-movement profile of less fluent oculomotor control and coordination of rapid sequential eye movements appears characteristic of individuals who perform poorly at RAN and serial reading tasks, including showing a greater number of fixations, shorter eye voice span (the lead in the position of the eyes compared to the position of the item being spoken), and more refixations [7, 12–17]. Considering the links between RAN and complex language-related skills, and RAN’s connection with known neural networks associated with language [9, 10, 18], studying gaze-language coordination during RAN in ASD and among first-degree relatives holds the potential to reveal basic language-related processes (and associated neural networks) underlying complex clinical features of ASD and reflecting genetic liability.

Impairments in RAN have been reported in ASD, as revealed through slower naming time and more frequent errors [1, 2]. Clinically unaffected first-degree relatives also show subtle differences in RAN that are most notable among parents with the BAP [2]. In the only prior study examining simultaneous vocalization and gaze during RAN in ASD, Hogan-Brown and colleagues reported longer naming times and reduced eye-voice span (EVS) in both ASD and among siblings, with a step-wise pattern where individuals with ASD evidenced the least fluency, followed by their siblings, then controls, suggesting that EVS may indicate a graded pattern of expression of genetic liability to ASD [1].

Eye-voice span reflects the fluent coordination of basic components of language (e.g., syntactic and semantic processing [19]). It can also reveal the automaticity of the coordination of sound, visual information, and linguistic processes, and the degree to which such coordination constitutes an effortless as opposed to resource-demanding process [7, 20–23]. In other words, automaticity of a skill is an established neurocognitive property related to learned behaviors, and which reflects the coordination of different components of that skill working together smoothly, accurately, and quickly. This coordination improves as typical development progresses [6]. Importantly, reduced automaticity may result in fewer neurocognitive resources available to support higher-level language processing [8] such as narrative production or pragmatic (social) language, which are commonly impaired in individuals with ASD [24–28]. Here, we examined EVS in ASD and added a more comprehensive range of gaze variables including refixations (perseverations and regressions) and total number of fixations as additional indices of language processing fluency. These additional measures allowed for fine-grained analysis of the coordination in time of visual and vocal processes and cognitive resources supporting RAN [29]. We also examine for the first time these gaze variables among parents with and without the BAP, and how their performance may relate to their children’s language and gaze coordination during RAN to assess familiality of RAN to ASD. Finally, we explored whether gaze and language coordination during RAN might relate to broader language and clinical-behavioral features in ASD and the BAP.

## Methods

### Participants

Participants included 51 individuals with ASD and 45 controls. A subset of these participants (17 ASD and 20 controls) were included in a previous study of more limited RAN measures in ASD [1]. The parent-ASD (*n* = 133) and parent-control (*n* = 58) groups were not included in any prior studies of RAN (see Table 1 for sample characteristics). There were 43 dyadic pairs (parent-child) in ASD families and 20 control dyads. There were 45 spousal pairs in the parent-ASD group and 6 pairs in the parent-control group. ASD and control families were recruited through study advertisements distributed to ASD clinics and advocacy organizations, participant registries, and word of mouth. Inclusionary criteria for all participants included having a minimum verbal and full-scale IQ (FSIQ) of 80, being a native speaker of English, and no visual impairment(s) or color blindness, no history of dyslexia or brain injury, and no known genetic syndrome associated with ASD or major psychiatric disorder (i.e., bipolar, schizophrenia, and related psychotic disorders). One parent of an individual with ASD was found to have strabismus/nerve damage. Her data were examined in relation to group means, and eye movement videos were analyzed, which revealed no obvious deviations. As such, her data were retained. Control participants were excluded if they had a family history of ASD, dyslexia, or language-related delays. Parents were included in the parent-ASD group if they had at least one child with idiopathic ASD. Those in the parent-control group had no personal or family history of ASD or related genetic disorders (e.g., fragile X syndrome). Full-scale, verbal, and performance IQ were assessed using the Wechsler Abbreviated Scale of Intelligence (WASI) [30] for all participants (see Table 1).

**Table 1:** Sample Characteristics

	Control Group	ASD Group			Group Comparisons		
	(*n* = 45; 23/22 M/F)	(*n* = 51; 42/9 M/F)					
	M (SD)	M (SD)			t	df	*p*
Age (years)	18.9 (5.8)	17.8 (8.5)			0.76	94	0.45
Full IQ	117.62 (12.37)	105.86 (12.77)			4.57	94	< 0.001
Performance IQ	113.76 (14.5)	104.90 (14.5)			2.97	94	0.004
Verbal IQ	118.82 (11.7)	105.82 (14.18)			4.86	94	< 0.001
Narrative ability (LSA)	0.50 (0.09)	0.40 (0.11)			3.96	60	< 0.001
ADOS^b^							
Social Affect Severity Score	–	6.4 (2.1)			–		
RRB Severity Score	–	7.0 (2.5)			–		
Total Severity Score	–	6.7 (2.3)			–		
ADI-R							
IS factor sum	–	0.93 (0.63)			–		
RSM factor sum	–	0.54 (0.44)			–		
	Parent-control Group	Parent-ASD Group			Group Comparisons between Parent-control and Parent-ASD Groups Overall		
	(*n* = 58; 22/36 M/F)	(*n* = 133; 50/83 M/F) (*n* = 62 BAP+; *n* = 66 BAP-)					
	M (SD)	M (SD)			t	df	*p*
		Overall	BAP(+)	BAP(−)			
Age (years)	41.2 (10.4)	46.2 (8)	47.0 (7)	45.3 (8.5)	−3.26	88^a^	0.002
Full IQ	114.4 (11.7)	111.9 (11.32)	112.5 (11.3)	111.4 (11.2)	1.38	189	0.17
Performance IQ	114.6 (13.1)	110.6 (11.54)	110.9 (11.2)	110.6 (11.7)	2.12	189	0.04
Verbal IQ	110.7 (12.9)	110.5 (11.61)	111.3 (12.5)	109.6 (10.8)	0.07	189	0.95
Narrative ability (LSA)	0.50 (0.09)	0.46 (0.11)	0.46 (0.12)	0.46 (0.10)	2.62	132^a^	0.01

*IS* Insistence on Sameness, *IQ* Intelligence Quotient, *LSA* Latent Semantic Analysis, *RRB* Restricted and Repetitive Behavior, *RSM* repetitive sensorimotor.

^a^Equal variance not assumed in statistical t-test

^b^Comparison severity score labels are as follows: 0–2 = “minimal-to-no evidence”, 3–4 = “low”, 5–7 = “moderate”, 8–10 = “high”

All study procedures were approved by the University of North Carolina at Chapel Hill and Northwestern University’s Institutional Review Boards and written informed consent/assent were obtained for all participants.

### Assessment of ASD symptoms

Diagnosis of ASD was confirmed and clinical symptom severity was assessed for individuals in the ASD group with research-reliable or intra-lab-reliable scoring and administration of the Autism Diagnostic Observation Schedule-General or 2nd Edition (ADOS) [31, 32] or the Autism Diagnostic Interview-Revised (ADI-R) [32–34]. Additionally, following prior work [35, 36], insistence on sameness and repetitive sensorimotor behavior current factor scores were computed from the ADI-R.

### Assessment of the broad autism phenotype in the parent-ASD group

Social and rigid personality features of the broad autism phenotype (BAP) were assessed among parents of individuals with ASD using the Modified Personality Assessment Scale-Revised (MPAS) [37], a semi-structured interview designed to elicit information about individuals’ personality features that has been used extensively in studies of the BAP [3]. Questions regarding social personality characteristics of the BAP tap interest and participation in social relationships. The rigid BAP personality feature is assessed through questions focused on the importance of and adherence to routines and organizational styles. Consistent with prior studies [3, 38, 39], coders blind to participant family diagnosis completed ratings from videos. Scores included 0 (trait absent), 1 (possibly present), and 2 (trait definitely present). Individuals were conservatively characterized as BAP(+) only if they scored a 2 on the Social or Rigid traits, and as BAP(−) if scoring < 2 across domains. These personality features are thought to mirror in quality the social and repetitive behavior domains of impairment in ASD and have been shown to reliably distinguish ASD relatives from controls [3, 39] and relate to both language (including RAN) and social cognitive skills in parents [2, 38].

### Design and stimuli

Participants completed a Rapid Automatized Naming (RAN) task from the Comprehensive Test of Phonological Processing (CTOPP) [40], which included two runs for each of four stimulus types (colors, letters, numbers, and objects—in order). Participants were instructed to rapidly and accurately name the stimuli in each row from left to right. Each run consisted of naming an array of 36 items (nine different items, randomly presented and repeated in four rows) on a 17-in. TFT LCD monitor (1280 × 1024 resolution) placed 18–24 in. away from the participant [1]. Before each condition, all participants completed a practice trial of the nine symbols included in the task to ensure mastery of task instructions and consistency of labels for each item. A Tobii T60 eye tracker (Tobii Technology AB, Danderyd, Sweden) was used to measure gaze coordinates at a rate of 60 Hz. Eye gaze was calibrated using a standard 5-point grid prior to the task. According to the manufacturer’s specifications, this device has a typical accuracy of 0.5° of visual angle. Participants were recalibrated following any large movements during calibration.

### Data processing

#### Vocal responses

The Penn Phonetics Lab Forced Aligner, an automatic and forced phonetic alignment toolkit that synchronizes phonetic transcriptions with speech signals [41], was used to mark the onset and offset of articulation of each item. Subsequently, the onset and offset boundaries of utterances were checked by trained coders who were blind to participant diagnostic status. Any errors in the marking or deviations from the expected names of the items were manually corrected by coders to reflect the participant’s actual response, including marking unexpected responses as errors [1, 7].

#### Gaze

An area of interest (AOI) for each item was operationally defined as a region extending vertically and horizontally from the middle of each item to the midpoint between each neighboring item. AOI size was consistent with that defined in Hogan-Brown et al. [1]. Fixations were therefore assigned to an AOI based on their spatial coordinates and consecutive fixations within the same AOI were pooled and used for additional variables. Fixations less than 80 ms were excluded from analyses, as these are typically associated with tracker error [29]. Runs with track loss of > 35% of total fixation duration within an individual run were also excluded from analyses. Additionally, minimum and maximum values for number of fixations per run were established based on outliers (> 2.5 SD above mean) and data distributions as an additional quality control measure (15 and 50 for letter/number trials, and 20 and 55 for color/object trials, respectively, due to the larger number of fixations observed during color/object trials). On average, 2.2% and 8.8% of runs were excluded for track loss due to track duration for the control group and ASD group, respectively. T-tests revealed no significant group difference on the proportion of mean trials excluded (*t*(23) = .46, *p* = .65). Averages of 3.9% and 6.7% of trials were omitted for the parent-ASD and parent-control groups, respectively. *T*-tests revealed no significant differences in track loss between parent groups (*t*(34) = .15, *p* = .88), nor any significant differences in track loss between BAP(+) and BAP(−) parents (*t*(18) = .49, *p* = .44). Eye movement data and vocal responses were aligned based on the start and ending time stamps for each trial.

Vocal responses and associated eye movements for the first two items and the last four items of each array were excluded from all eye tracking analyses because of difficulties in interpreting eye movements in relation to those items, such as mistargeting the long saccades back to the beginning of a row (see Gordon & Hoedemaker, 2016 for a more detailed description of data processing procedures [7]).

### Analysis procedures for RAN

For each variable of interest, both runs per condition (stimulus type) were averaged to produce one mean variable per condition. Further, consistent with prior work [1, 2, 6, 7, 42], conditions were averaged to assess symbolic (letter/number) performance vs. non-symbolic (object/color), given that these conditions tap different levels of language automaticity [7, 16].

#### Naming performance

##### Overall naming time

Naming time was calculated as the time between the onset of the first articulation to the offset of the final (36th) item on each run. Whereas overall naming time analysis included erroneous responses and pauses, naming time for correct trials only and naming time without pauses or errors showed similar findings and were significantly correlated with *overall naming time* between and within groups. As such, erroneous responses and pauses were not likely to have contributed significantly to overall completion time differences.

##### Frequency of errors

All errors and self-corrections observed during RAN were totaled, including substitutions (e.g., saying “green” instead of “red”), omissions, or repetitions.

#### Eye movement

##### Eye-voice span (EVS)

EVS was defined as the number of items ahead the eye gaze was compared to the voice at the onset of each vocal response. EVS only included correct responses and data were omitted for two subsequent responses following errors, as eye movement patterns are often disrupted during errors due to regressions and self-corrections. Any EVS < 0 or > 5 for a given item was excluded from analyses, as such values likely reflect poor tracking and/or off-task behavior.

##### Number of fixations overall

This was defined as the total number of fixations made during the whole run.

##### Refixations

Refixations were defined as fixations to either previously fixated stimuli (i.e., regressions) or within the same stimuli currently being fixated (i.e., perseverations).

### Complex language use

We assessed participants’ narrative ability, a complex social communicative skill that is impacted in ASD and the BAP [24, 25, 43, 44]. Narrative language was assessed through latent semantic analysis (LSA) applied to participants’ narrative descriptions of six illustrated stimuli from the Thematic Apperception Test (TAT) [45] used previously to elicit narratives [26, 46, 47]. LSA is a computational linguistic tool whereby written text is automatically compared to a large corpus of language samples by extracting semantic content from the narrative, filtering out high-frequency words, plotting this information into a vector space, selecting the “prototypical narratives”, and assigning a quantitative measurement of similarity for each individual’s narrative to this prototype, ranging from − 1 (more dissimilar) to 1 (identical)—i.e., higher scores indicate better narrative quality, which is more similar to prototypical narratives. This method has been used to evaluate narrative quality in typical and atypical development and has been validated across different narrative contexts in ASD and related psychiatric disorders [24, 26, 48]. Narratives were transcribed by transcribers blind to group status and trained to at least 80% word-level reliability. A second independent transcriber assigned word and utterance segmentation.

### Statistical analysis plan

#### Group comparisons

Differences in RAN performance were assessed using a series of 2 × 2 (group × condition) repeated measures analysis of variance (ANOVAs) or ANCOVAs, separately for ASD vs. control, and for parents (parent-ASD group vs. parent-control group), and 3 × 2 repeated measures ANOVAs or ANCOVAs to explore differences by BAP status in the parent-ASD group versus controls. To account for significant differences in FSIQ between ASD and control groups, FSIQ was added as a control variable for all analyses of the ASD and control groups. Additionally, since age significantly correlated with RAN naming time and error variables in the ASD and control groups, age was covaried in these analyses. For parents, only naming time and error analyses controlled for FSIQ, as IQ was not related to eye movement variables. Age was not associated with RAN variables in parents and therefore was not added as a covariate. Significance level was set at *p* < .05 for all models, but *p* values of < .008 are noted as withstanding Bonferroni correction for multiple comparisons. Planned pairwise comparisons for groups are also reported. Simple effect *t*-tests were conducted when group × condition interactions were significant, and degrees of freedom were corrected to account for non-equality of variances. Since assumptions of ANOVA (e.g., normality) were not met for most variables, analyses were followed by non-parametric tests (Mann-Whitney *U*), which replicated all findings. Finally, because a subgroup of ASD and ASD-control participants overlapped with participants included in a prior report [1], we replicated analyses of RAN variables previously reported (naming time, errors, and EVS) with these individuals removed.

We predicted that individuals with ASD would demonstrate poorer RAN performance and associated differences in eye movements (shorter EVS, greater number of fixations and refixations) compared to controls, and that qualitatively similar but more subtly expressed differences would be evident in parents of individuals with ASD. Associations between RAN naming and frequency of errors and gaze variables were also examined using Pearson correlations.

#### Group comparisons in parents nested within families

Given that spousal pairs were included in both parent groups and considering evidence of assortative mating in cognitive ability, reading, and within families of individuals with ASD [49–51], we also examined spousal similarity in RAN as an index of potential assortative mating within families. A series of mixed effects linear regression models investigating all RAN variables for parents only were fitted using the lmer package [52] for R statistical software. All models included fixed effects of group, condition, and their interaction. For random effects, model 1 had individuals nested within families while model 2 did not. We explored model fit to assess whether model 1 and model 2 were significantly different from one another (i.e., indicating spousal resemblance, and potential influence of assortative mating or other environmental influences). Model fit was assessed using the chi-square (*χ*^2^) statistic.

#### Sex differences in RAN performance and gaze

Post hoc analyses explored group × sex interactions across all RAN variables separately for the ASD and control groups, and parent-ASD and control groups (and by BAP status).

#### Associations with clinical-behavioral measures

To examine associations between RAN performance and ASD symptoms and narrative quality, partial correlations were conducted, controlling for demographic variables that differed between groups or correlated with RAN. Specifically, age and IQ were controlled in analyses with RAN naming time in the ASD and control groups, and FSIQ only in partial correlations involving eye movement variables in these groups. In parents, full-scale IQ was a control variable for naming time correlations.

We predicted that naming time and refixations would be associated with the restricted and repetitive behavior domain of ASD, due to the conceptual links between these features (e.g., perseverative tendencies and challenges with attentional disengagement in ASD) [53, 54]. We expected that reduced EVS and longer naming times (indicative of expending greater cognitive resources) would correlate with reduced narrative competence [24–26, 55]. Because the average number of errors per RAN trial was low across groups, error rates were not included in correlational analyses.

#### Parent-child correlations

To examine familiality of RAN in ASD, we applied Pearson’s correlation coefficient by parent sex (mother or father) among the intact parent-child dyads. We examined parent-child correlations for the same RAN variable between dyads. We also examined correlations between parent RAN variables and child ASD symptom severity (ADOS and ADI-R), IQ, and narrative ability, as a means to explore indices that are often used to subgroup individuals with ASD and more broadly reflective of ASD symptom severity. Following Pearson’s correlations, we used Fisher’s *z* tests (i.e., conducted *r*-to-*z* transformations), to empirically test whether any detected parent-child associations were stronger in mother- versus father-child dyads.

Given the exploratory nature of correlational analyses, these were not corrected for multiple comparisons. While we acknowledge that this increases the false discovery rate, our interpretation of the correlations was primarily reliant on close inspection of the overall pattern, including the size and direction of the correlations, and less reliant on inference based simply on significance testing.

## Results

**Fig. 1:**
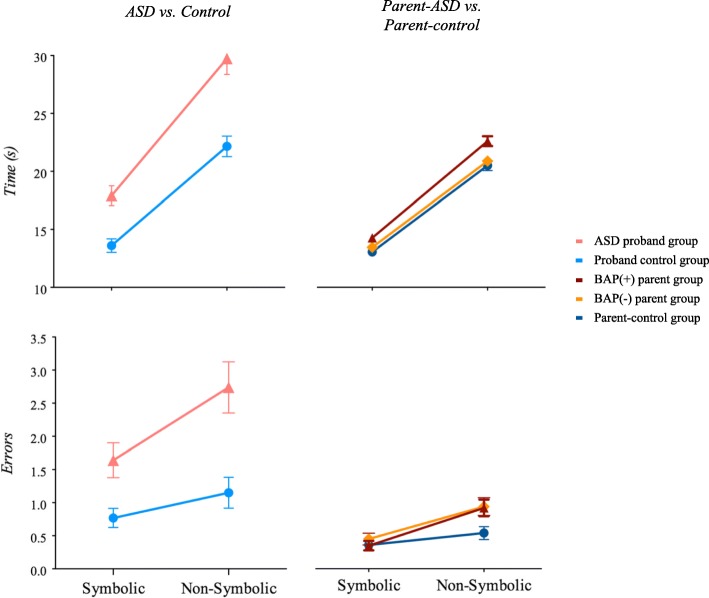
Figures display naming time and error rate in the ASD vs. control groups on the left panel, and parent-ASD group vs. parent-controls on the right panel

**Fig. 2:**
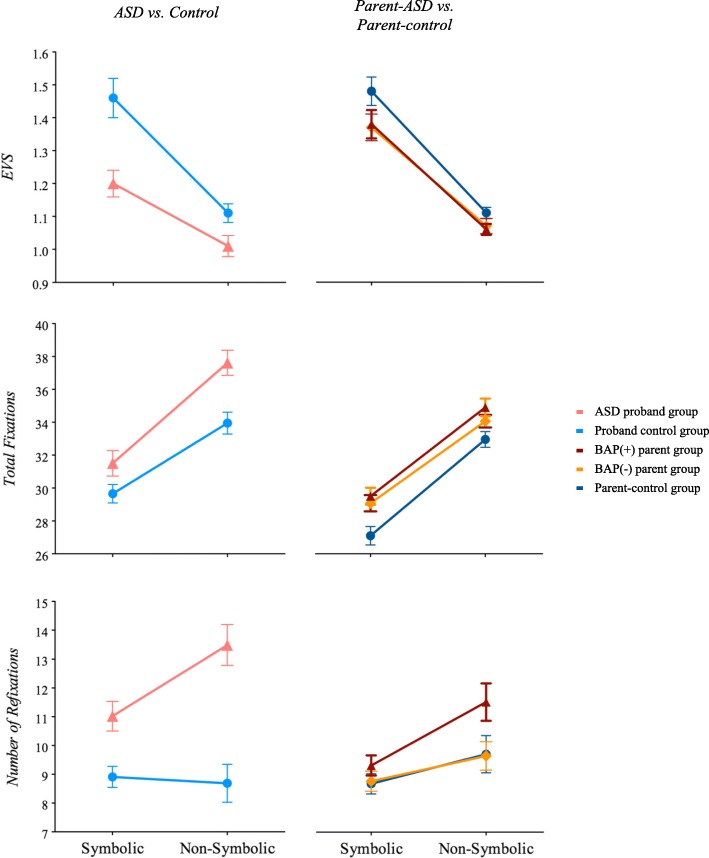
Figures display eye-voice span, total fixations, and refixations in the ASD vs. control groups on the left panel, and parent-ASD group vs. parent-controls on the right panel

Descriptive statistics and group statistical comparisons are presented in Table 2 and Fig. 1 (Naming performance) and Fig. 2 (Eye movement during RAN), and summarized below.

**Table 2:** Summary of Results

	Control Group		ASD Group		Main Effect of Group				Main Effect of Condition^a^				Group X Condition^a^ Interaction			
	Symbolic	Non-Symbolic	Symbolic	Non-Symbolic												
	M (SD)	M (SD)	M (SD)	M (SD)	F	df	*p*	η^2^	F	df	*p*	η^2^	F	df	*p*	η^2^
Naming Performance																
Overall Naming Time	13.61 (3.91)	22.16 (5.93)	17.91 (6.09)	29.74 (9.74)	**15.33**	**1, 92**	**< 0.001** ^*^	**0.14**	**8.78**	**1, 92**	**0.004** ^*^	**0.09**	**9.66**	**1, 92**	**0.003** ^*^	**0.10**
Errors	0.77 (0.97)	1.15 (1.57)	1.64 (1.87)	2.74 (2.76)	**7.95**	**1, 92**	**0.006** ^*^	**0.08**	**4.75**	**1, 92**	**0.032**	**0.05**	2.04	1, 92	0.157	0.02
Eye Movement																
Eye-voice Span	1.46 (0.4)	1.11 (0.19)	1.2 (0.29)	1.01 (0.23)	**12.29**	**1, 93**	**0.001** ^*^	**0.12**	1.61	1, 93	0.207	0.02	**6.84**	**1, 93**	**0.01**	**0.07**
Total Fixations	29.66 (3.74)	33.95 (4.44)	31.51 (5.55)	37.62 (5.47)	**5.46**	**1, 93**	**0.022**	**0.06**	2.51	1, 93	0.117	0.03	2.08	1, 93	0.153	0.02
Refixations	8.91 (2.47)	8.69 (4.44)	11.02 (3.7)	13.49 (5.07)	**13.96**	**1, 93**	**< 0.001** ^*^	**0.96**	0.73	1, 93	0.40	0.14	**8.00**	**1, 93**	**0.006** ^*^	**0.80**
	Parent-control Group		Parent-ASD Group		Main Effect of Group				Main Effect of Condition				Group X Condition Interaction^a^			
	Symbolic	Non-Symbolic	Symbolic	Non-Symbolic												
	M (SD)	M (SD)	M (SD)	M (SD)	F	df	*p*	η^2^	F	df	*p*	η^2^	F	df	*p*	η^2^
Naming Performance																
Overall Naming Time	13.05 (2.2)	20.51 (3.32)	13.89 (2.44)	21.78 (3.32)	**5.95**	**1, 188**	**0.016**	**0.03**	**8.60**	**1, 188**	**0.004** ^*^	**0.04**	0.91	1, 188	0.342	0.01
Errors	0.36 (0.5)	0.54 (0.75)	0.41 (0.65)	0.94 (1.06)	**3.26**	**1, 188**	**0.041**	**0.02**	**4.66**	**1, 188**	**0.032**	**0.02**	3.83	1, 188	0.052	0.02
Eye Movement																
Eye-voice Span	1.48 (0.33)	1.11 (0.13)	1.37 (0.33)	1.06 (0.16)	**5.13**	**1, 189**	**0.025**	**0.03**	**234.01**	**1, 189**	**< 0.001** ^*^	**0.55**	2.33	1, 189	0.129	0.01
Total Fixations	27.1 (4.25)	32.96 (3.64)	29.39 (4.24)	34.54 (3.8)	**13.43**	**1, 189**	**< 0.001** ^*^	**0.07**	**255.13**	**1, 189**	**< 0.001** ^*^	**0.57**	1.05	1, 189	0.306	0.01
Refixations	8.68 (2.65)	9.71 (4.91)	9.09 (2.93)	10.58 (4.67)	1.50	1, 189	0.22	0.23	**15.03**	**1, 189**	**< 0.001** ^*^	**0.97**	0.52	1, 189	0.47	0.11

Bold indicates significance *p* < .05.

^a^Condition refers to symbolic (letter and number) and non-symbolic (object and color) RAN conditions

^*^indicates significance following Bonferroni corrected significance value *p* ≤ .008

### Naming performance

#### Naming time

##### ASD vs. control

The ASD group had slower times overall, and both groups exhibited longer naming times during the non-symbolic than symbolic conditions. A group × condition interaction revealed that the ASD group showed significantly slower naming times than controls, particularly during the non-symbolic trials (planned comparisons symbolic *t*_(94)_ = − 4.05, *p* < .0001; non-symbolic *t*_(83.96)_ = − 4.67, *p* < .0001).

##### Parent-ASD vs. parent-control

The parent-ASD group was slower overall, but findings did not meet the threshold for significance after correction for multiple comparisons. Both parent groups took longer to name non-symbolic items. This difference was driven by the BAP(+) group, who showed significantly slower naming times across conditions compared to BAP(−) parents and controls (overall model, (*F*_(2,182)_ = 8.16, *p* < .0001); planned comparison for BAP(+) vs. control and BAP(+) vs. BAP(−) *ps* < .01. BAP(−) parents did not differ from controls (*p* = .53).

#### Frequency of errors

##### ASD vs. control

Both groups made significantly more errors during the non-symbolic condition, though not significant after correction for multiple comparisons, and the ASD group made more errors than controls overall.

##### Parent-ASD vs. parent-control

Parents made very few errors overall (averaging < 1); both groups committed more errors during non-symbolic than symbolic trials (though like the ASD and control groups, this did not withstand correction for multiple comparisons). While no group differences emerged overall, the parent-ASD group made more errors during the non-symbolic condition relative to controls (planned comparison symbolic *t*_(189)_ = − 4.46, *p* = .65; non-symbolic *t*_(150.67)_ = − 2.94, *p* < .01). No significant differences were detected based on BAP status (overall model, (*F*_(2,182)_ = 2.06, *p* = .13; planned comparison for BAP(+) vs. BAP(−) *p* = .63, BAP(+) vs. control and BAP(−) vs. control *ps* > .05).

#### Re-analysis of RAN performance and EVS in a sub-group

##### ASD vs. control

Group effects (after removing the subgroup of overlapping participants in a prior report—i.e., 17 ASD and 20 controls) [1] for naming time and errors remained consistent with the full sample (*F*_(1,55)_ = 7.79, *p* < .01; *F*_(1,55)_ = 3.97, *p* = .05, respectively). In contrast, EVS differences were no longer significant (*F*_(1, 55)_ = 1.31, *p* = .26).

### Eye movement during RAN

#### Eye-voice span (EVS)

##### ASD vs. control

The ASD group had significantly shorter EVS compared to controls, and the difference between groups was larger during the symbolic trials (planned comparisons symbolic *t*_(94)_ = 3.66, *p* < .0001; non-symbolic *t*_(94)_ = 2.37, *p* < .05).

##### Parent-ASD vs. parent-control

While not meeting Bonferroni corrected cut-off for significance, the parent-ASD group had a shorter EVS compared to controls, and all parents had a shorter EVS during non-symbolic trials. Group effects were not driven by BAP status (overall model *F*_(2,183)_ = 2.45, *p* = .09; planned comparison for BAP(+) vs. BAP(−) *p* = .91, planned comparison for BAP(+) vs. control and BAP(−) vs. control *p*s > .05).

#### Number of fixations

##### ASD vs. control

The ASD group made more fixations than controls across conditions, though not significant after Bonferroni correction.

##### Parent-ASD vs. parent-control

The parent-ASD group made a significantly larger number of fixations overall. Both groups made more fixations overall during non-symbolic vs. symbolic conditions. Differences were not driven by BAP status, with similar patterns observed in both the BAP(+) and BAP(−) groups (overall model *F*_(2,183)_ = 7.15, *p* = .001; planned comparison for BAP(+) vs. BAP(−) *p* = .27, BAP(+) vs. control and BAP(−) vs. control *p*s < .01).

#### Refixations

##### ASD vs. control

Refixations occurred significantly more often in the ASD group compared to controls, particularly during the non-symbolic conditions (planned comparisons symbolic *t*_(87.71)_ = − 3.33, *p* < .01; non-symbolic *t*_(94)_ = − 4.90, *p* < .0001).

##### Parent-ASD vs. parent-control

Both groups made more refixations during the non-symbolic condition. The BAP(+) parents made significantly more refixations than the parent-control group and BAP(−) parents, (*F*_(2,183)_ = 2.92, *p* = .06; planned comparison for BAP(+) vs. control and BAP(+) vs BAP(−) *p*s < .05), though findings did not reach significance after Bonferroni correction. BAP(−) parents did not differ from controls (*p* = .98).

### Group comparisons in parents nested within families

Mixed effects linear regressions tested the influence of within-family clustering (i.e., spousal similarity) of RAN skills by comparing model fit, via a chi-square difference test, of models with and without parents nested within families as a random effect. While variances for the within-family clustering were large, these analyses indicated that they did not significantly impact the fixed effects of group in these models for any RAN variable: naming time (*χ*^2^(1) = .09, *p* = .77), frequency of errors (*χ*^2^(1) = 1.37, *p* = .24), EVS (*χ*^2^(1) = .007, *p* = .93), total number of fixations (*χ*^2^(1) = 0, *p* = 1.0), and total number of refixations (*χ*^2^(1) = .31, *p* = .58).

### Sex differences in RAN performance and gaze

No group × sex interactions emerged across any RAN or gaze variables for the ASD vs. control groups (*F*s_(1,91)_ < .55, *p*s > .47), parent-ASD vs. control groups (*F*s_(1186)_ < 1.39, *p*s > .15), or by BAP status (*F*s_(1179)_ < 1.21, *p*s > .30).

### Associations between eye movement and RAN performance

#### ASD vs. control

In both groups, larger EVS during symbolic conditions was correlated with faster naming time across conditions (*r*s > − .36, *p*s < .01). Total fixations and refixations during both symbolic and non-symbolic conditions were positively correlated with naming time in both groups (*r*s > .59, *p*s < .001; *r*s > .29, *p*s < .05, respectively). Refixations during non-symbolic conditions were also positively associated with frequency of errors in the control group (*r* = .37, *p* < .05).

#### Parent-ASD vs. parent-control

In both groups, larger EVS during both conditions was correlated with faster naming time (*r*s > −.40, *p*s < .01). Total fixations and refixations in both conditions were positively correlated with naming time in both groups (*r*s > .55, *p* < .001 and *r*s > .27, *p*s < .05, respectively). These correlations held only for the BAP(+) group in subgroup analyses, such that during both conditions, faster naming time was correlated with greater EVS (*r =* −.50, *p* < .001), and fewer total fixations and refixations (*r* = .53, *p* < .001 and *r* = .41, *p* < .01, respectively)*.*

### Clinical-behavioral correlates of RAN ability

**Fig. 3:**
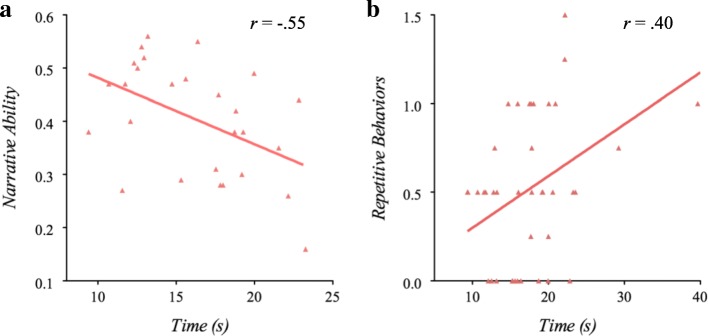
Clinical-behavioral correlates of RAN ability. **a** Longer RAN naming time was associated with lower narrative ability among individuals with ASD. **b** Longer RAN naming time was correlated with increased severity of restricted and repetitive behaviors in ASD

In the ASD group, longer naming times across conditions were associated with poorer narrative ability *(*Fig. 3a; *r*s > −.55, *p*s < .01). Additionally, greater fixations and refixations during the symbolic condition were correlated with poorer narrative quality (*r* = −.55, *p* < .01; *r* = .40, *p* < .05, respectively). Longer naming times across conditions were also associated with higher scores of restricted and repetitive behaviors (RRBs; particularly the repetitive sensorimotor domain) on the ADI-R *(*Fig. 3b; *r*s > .35, *p*s < .05). Greater refixations (in particular, regressive fixations) were also correlated with higher repetitive sensorimotor factor scores on the ADI-R (*r* = .40, *p* = .01).

No correlations with narrative ability emerged in the parent-ASD or either control group.

### Parent-child correlations as an index of familial effects in RAN

Parent-child correlations revealed that parent RAN naming time, EVS, and refixations (during non-symbolic conditions) were correlated with greater severity of RRBs in individuals with ASD (particularly the insistence on sameness ADI-R factor) (*r* = .46, *p* < .01; *r* = −.41, *p* < .05; *r* = .43, *p* < .05, respectively). Correlations for naming time and EVS with RRBs were significant for mothers only (*r* = .46, *p* < .01 and *r* = −.41, *p* < .05, respectively), with both mothers and fathers showing relationships with child RRBs for refixations (regressions in particular; mothers *r* = .43, *p* < .05; fathers *r* = .54, *p* < .01).

**Fig. 4:**
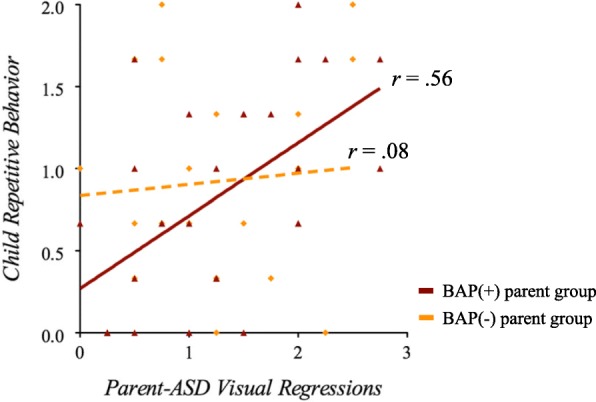
Parent-child correlations in families of individuals with ASD. Greater rates of visual regressions in BAP(+) parents only (mothers and fathers) were associated with increased severity of restricted and repetitive behaviors in their children with ASD. This pattern was not observed for BAP(−) parents

When segregating by BAP status, associations with child RRBs were observed in BAP(+) mothers for naming time (*r* = .72, *p* < .01) and in BAP(+) mothers and fathers for refixations (regressions in particular; *r* = .56, *p* < .01), but not for the BAP(−) subgroup (*r* = .08, *p* = .74) (Fig. 4). Positive associations were also detected between EVS (during symbolic conditions) in parents overall and in mothers with the BAP in particular with child full-scale IQ (*r*s > .27, *p*s < .05). No associations emerged between parent RAN performance or gaze and child RAN variables, social communication, and narrative ability.

Fisher’s *z* transformation tests revealed no significant differences in the strength of parent-child correlations between mother- versus father-child dyads across reported RAN indices (naming time *z* = .45, *p* = .65; EVS *z* = − 1.48, *p* = .14; regressions *z* = −.49, *p* = .62). In terms of BAP(+) associations, Fisher’s *z* tests resulted in non-significant differences between reported mother- and father-child correlations (naming time *z* = 1.10, *p* = .27; regressions *z* = −.19, *p* = .85). However, significant sex-specific differences for BAP(+) mother and BAP(+) father associations with their child’s full-scale IQ emerged (EVS *z* = 2.39, *p* < .05).

## Discussion

This study analyzed gaze and language during rapid automatized naming (RAN) among individuals with ASD and their parents to investigate how the basic language and related neurocognitive processes indexed by RAN may relate to the clinical-behavioral features of ASD and the broad autism phenotype (BAP). Examining co-occurring vocalizations and eye movement allowed for fine-grained analysis of the coordination in time of visual and vocal processes and cognitive resources supporting RAN. Findings revealed parallel differences along gaze and language measures of RAN in the ASD and parent-ASD groups (though more mildly expressed among parents), with the BAP(+) subgroup driving differences in naming time and refixations. We also detected associations between RAN and clinical-behavioral features in ASD. Together, findings suggest that these RAN-related skills potentially constitute genetically meaningful features or candidate language-related ASD endophenotypes. Given the emerging literature on the distributed neural correlates of RAN [6, 10, 18, 56], and findings of high heritability and significant genetic linkage and association of RAN with a number of genomic regions [57–61], RAN may be an important target for future investigations examining neural and molecular genetic correlates in ASD.

Findings of slower naming times and increased errors, as well as a shorter eye-voice span (i.e., lead in the eyes compared to the voice; EVS) in ASD are consistent with prior work [1], although EVS group differences were attenuated after removing the sub-group of participants that overlapped with this prior report. Whereas the control group’s gaze tended to lead speech by almost two items [7], vocalization with gaze were much more tightly coupled in the ASD group. Given that EVS is a marker of fluency and coordination of linguistic and visual/attentional processes, and considering that shorter EVS was associated with longer naming time, the reduced EVS observed in the ASD group suggests less-efficient perceptual encoding and reduced automaticity overall [7, 20–23]. The ASD group also demonstrated greater fixation and refixation frequency during RAN (which also predicted longer naming times), suggesting that regressive or perseverative eye movements reflect processing disruptions impacting language processing [7]. This pattern is consistent with reports of increased perseverative eye movements in ASD across a number of different social and non-social visual attention tasks in ASD [53, 62–65] and may reflect more domain-general underlying deficits impacting a range of functions [5].

Notably, atypical gaze and naming patterns in the ASD group were related to clinical-behavioral features of ASD. Longer naming time, and higher total fixation and refixation rates were associated with lower narrative ability, and slower naming time was associated with more severe restricted and repetitive behaviors (RRBs). Associations between RAN-related gaze and vocalization patterns and narrative quality highlight how fundamental language processing disruptions might contribute to more widespread language impairments that characterize ASD [63]—i.e., when more effort is required for executing basic-level language processing, fewer resources are available for allocating to more complex language functions such as narrative (which is impaired in ASD) [24–26, 55]. Findings that slower naming and increased refixations related to more severe RRBs in ASD (in particular, lower-order motoric RRBs, as opposed to more cognitively based RRBs that fall under the insistence on sameness sub-category [35, 36]) may suggest a lower capacity to “reset” cognitive processes, resulting in “getting stuck” or perseverating [66] as a common underlying mechanism related to RRBs and language processing in ASD. It is also possible that disruptions in executive control, visuospatial processing, motor speed, and interference in visual to motor transformation processes may contribute to slower naming times and their relationship to RRBs in ASD. Interestingly, prior research has demonstrated shared common brain regions involved during RAN, and the neurocircuitry of RRBs and social communication in ASD and other language-related disorders (e.g., cerebellum and left-hemisphere language temporal areas) [6, 9, 10, 18, 67–69]. Together, evidence suggests that RAN taps general neuropsychological mechanisms implicated in a number of complex symptom domains in ASD, including language-related impairments and repetitive behaviors.

Many of the patterns observed in the ASD group were mirrored (though more subtly expressed) in the parent-ASD group, including slower naming time, increased errors, reduced eye-voice span, and increased frequency of fixations and refixations. Importantly, these differences persisted when accounting for spousal similarity, suggesting that findings were not accounted for by assortative mating or other relational factors. Differences in naming time and refixation frequency were driven by the subgroup of parents who exhibited the BAP, consistent with a prior study of RAN naming time in parents [2]. Perhaps surprisingly, differences in EVS, total number of fixations, and errors were not specific to the BAP and were observed among the parent-ASD group overall. Important to consider is that EVS, total fixations, and errors during RAN indices are associated with neurocognitive skills implicated in a number of language and learning disabilities. Such differences between controls and the parent-ASD group may therefore reflect genetic liability to language-related disorders more broadly. By contrast, refixations (and their impact on naming time) may relate more selectively to the perseverative tendencies that are more characteristic of ASD and the BAP (e.g., “rigid” personality styles). Independent associations between EVS and refixations with naming time (but non-significant relationships between EVS and refixations) further indicate potentially unique mechanistic processes within the context of RAN. It may be that in the BAP, a “double hit” of vulnerability exists within these two constructs. Whereas RAN differences observed among all parents (and also in ASD) could reflect neurocognitive underpinnings influenced by more general genetic factors not specific to ASD, those RAN differences more specific to the BAP and also linked with ASD symptomatology (RRBs, narrative impairment) could reflect genetic influences more specific to ASD. In line with this possibility, similar patterns have been documented in other domains (e.g., social cognition, face processing [38, 70, 71]), such that differences in tasks most closely conceptually related to the core symptoms of ASD were specifically observed in a subgroup of parents who displayed the BAP, and other skill differences were observed more broadly among all parents of individuals with ASD.

Examining parent-child correlations with RAN ability revealed relatively robust associations between parents’ RAN (naming time, EVS, and refixations) and RRBs in their children with ASD, supporting connections between RAN and RRBs detected within the ASD group. Interestingly, associations appeared to be driven by BAP(+) parents. Parents’ EVS was also related to child IQ and was most strongly observed among mothers with the BAP. Evidence that such parent-child correlations are most evident in families with BAP(+) may help inform patterns of inheritance of ASD candidate endophenotypes, and stratification of families into more etiologically homogeneous subgroups to facilitate studies of underlying biology.

Finally, consistent with reports indicating greater executive functioning requirements during color/object naming [1, 6, 16, 72], all groups performed worse during non-symbolic (vs. symbolic) conditions. That the ASD group showed even more difficulty during non-symbolic conditions compared to controls suggests inefficient recruitment of executive functions in this group. Conversely, *all* parents (parent-ASD and parent-control groups) demonstrated poorer performance during non-symbolic conditions—a finding that was unsurprising in parents who were not clinically impaired [7].

### Strengths and limitations

Strengths of this study include the relatively large sample size of parents, permitting extension of earlier findings among siblings of individuals with ASD to parents, both with and without the BAP, along with the examination of more extensive measures of eye movement than had been examined previously, and which disaggregate in novel ways in relationship to RAN (e.g., tapping into processing speed, efficiency/fluency, executive functions such as flexibility as they relate to language and clinical features in ASD and the BAP). This study also examines a range of clinical-behavioral correlates that are theoretically and conceptually related to RAN skills, where associations between eye-voice coordination reported in both the ASD and parent-ASD groups may help to inform the underlying mechanistic factors associated with complex clinical phenotypes that characterize ASD and the BAP.

Several limitations should also be considered and addressed in future work. First, given the promising findings reported in this study, future work should examine in greater detail potential clinical-behavioral correlates of RAN, beyond features of the ASD and the BAP and global indices of narrative ability assessed here. It will be important, for instance, to build on the present findings to examine more fine-grained measures of complex language and related cognitive domains to determine how the relatively widespread RAN differences documented here might relate to more complex skills in clinically unaffected individuals. Additionally, studies including larger samples of females with ASD are needed to examine potential sex-specific patterns of RAN in ASD, which were not apparent in the present sample (perhaps owing to the low number of females with ASD (*n* = 9)). Further, although analyses covaried for age in the ASD group, and age was not associated with RAN in parent groups, this study included a relatively wide age range in our ASD group. Given existing developmental patterns observed in RAN in typical development and dyslexia [11, 73], future studies should explore particular developmental periods to understand whether RAN-related skills relate to different aspects of language and cognitive challenges over the course of development in ASD. Finally, because we necessarily restricted our sample to participants with verbal and full-scale IQ greater than 80 (in order to investigate RAN performance in the absence of more widespread language impairments), RAN-related skills should be examined in lower functioning groups as well. To the extent that RAN taps broader executive skills important for verbal *and* nonverbal communication (e.g., joint attention, multi-sensory coordination), differences in RAN observed in the ASD and parent-ASD groups might have implications across the autism spectrum, including younger and minimally verbal individuals.

## Conclusions

In sum, results significantly extend prior reports of RAN differences in both ASD and parent groups by documenting parallel differences in gaze during RAN in ASD and among parents of individuals with ASD, and links to clinical-behavioral features in ASD and the BAP. Together, findings replicate prior reports that RAN-related skills are impacted in ASD and among first-degree relatives [1, 2] adding to existing evidence that key cognitive systems linked with relatively well-defined underlying neural substrates are affected by ASD genetic risk and observable in ASD, and (even if subtly) also among unaffected first-degree relatives. Specific associations between RAN and the BAP further highlight the quantifiable nature of RAN and its relevance for future studies incorporating genetically meaningful phenotypes in biological studies of ASD.

## Abbreviations

ADI-R: Autism Diagnostic Interview-Revised
ADOS: Autism Diagnostic Observation Schedule
ANOVA: Analysis of variance
AOI: Area of interest
ASD: Autism spectrum disorder
BAP: Broad autism phenotype
CTOPP: Comprehensive Test of Phonological Processing
EVS: Eye-voice span
FSIQ: Full-scale intelligence quotient
LSA: Latent semantic analysis
MPAS: Modified Personality Assessment Scale
RAN: Rapid automatized naming
RRB: Restricted and repetitive behaviors
TAT: Thematic Apperception Test
